# Produced water treatment by semi-continuous sequential bioreactor and microalgae photobioreactor

**DOI:** 10.1186/s40643-024-00775-3

**Published:** 2024-06-02

**Authors:** Nur Farahah Mohd Khairuddin, Nadeem Khan, Saravanan Sankaran, Wasif Farooq, Irshad Ahmad, Isam H. Aljundi

**Affiliations:** 1 Membranes and Water Security IRC, King Fahd University of Petroleum and Minerals (KFUPM), Dhahran, Saudi Arabia; 2 Bioengineering Department, King Fahd University of Petroleum and Minerals (KFUPM), Dhahran, Saudi Arabia; 3 Chemical Engineering Department, King Fahd University of Petroleum and Minerals (KFUPM), Dhahran, Saudi Arabia

**Keywords:** Produced water, Wastewater treatment, *Scenedesmus obliquus*, Activated sludge, Sequential batch reactor, Photobioreactor

## Abstract

Produced water (PW) from oil and gas exploration adversely affects aquatic life and living organisms, necessitating treatment before discharge to meet effluent permissible limits. This study first used activated sludge to pretreat PW in a sequential batch reactor (SBR). The pretreated PW then entered a 13 L photobioreactor (PBR) containing *Scenedesmus obliquus* microalgae culture. Initially, 10% of the PW mixed with 90% microalgae culture in the PBR. After the exponential growth of the microalgae, an additional 25% of PW was added to the PBR without extra nutrients. This study reported the growth performance of microalgae in the PBR as well as the reduction in effluent’s total organic carbon (TOC), total dissolved solids (TDS), electrical conductivity (EC), and heavy metals content. The results demonstrated removal efficiencies of 64% for TOC, 49.8% for TDS, and 49.1% for EC. The results also showed reductions in barium, iron, and manganese in the effluent by 95, 76, and 52%, respectively.

## Introduction

Produced water (PW) is water that emerges during crude oil extraction, and it often contains hazardous materials that can harm living organisms. Among the hazardous materials are Uranium-238 (^238^U) and Radium (^226^Ra), which can cause severe radioactive pollution (Wu et al. 2023). Furthermore, PW also contains heavy metals, hydrocarbons, organic matter, and high concentrations of salts, which can pose significant obstacles to its safe reuse in irrigation practices (Al-Ghouti et al. 2019). For instance, a study has reported on PW treatment in an oilfield in the Niger Delta region, highlighting the challenges and efforts to address these contaminants. The oilfield used a flotation system as its primary treatment, where bubbles adhere to pollutants, causing them to float to the water’s surface, where they are subsequently removed by skimming. However, analysis of the treated PW showed that it did not meet the permissible limits (Table 1). The contaminants in the treated PW included BTX, phenol, oil, and grease (Amakiri et al. 2023).

**Table 1:** Summary of treated produced water (PW) characteristics from Niger Delta region (Amakiri et al. 2023)

Contaminants	(mg/L)	Permissible limit by Department of Petroleum Resources (DPR) (mg/L)
BTX	0.19–0.42	0.2
Dissolved oxygen	14–33	10
Total suspended solid (TSS)	87–216	50
Phenol	1–1.2	0.5
Oil and grease	15–28	20
Lead (Pb)	0.05–0.23	0.05
Nickel (Ni)	1.4–3	1.0
Iron (Fe)	1.02–1.27	1.0

Given these shortcomings, it is essential to consider other methods for treating PW. PW treatment can be divided into physical, chemical, and biological methods. Physical treatments include filtration, flotation, and electrolysis, while chemical treatments involve chemical oxidation and precipitation techniques. Biological treatments consist of activated sludge and microalgae-based methods (Al-Kaabi et al. 2021). Integrating multiple methods yields better results, as each method has its disadvantages. For example, membrane filtration is known for being prone to fouling, while adsorption methods require frequent absorbance replacement and regeneration (Abbas et al. 2021). There have been a few research on incorporating adsorption techniques with advanced oxidation systems to treat PW, but the research needs further study for its cost analysis (Alomar et al. 2022).

Unlike adsorption techniques that often require chemicals to regenerate or replace adsorbents, microalgae cultivation generally requires fewer chemicals. In fact, microalgae are effective at removing nutrients like nitrogen and phosphorus, which is rarely addressed in adsorption. The utilization of microalgae for wastewater bioremediation treatment involves three mechanisms: absorption of contaminants by microalgae, accumulation of pollutants within microalgae cells, and degradation of pollutants through the metabolic activities of microalgae (Abdelfattah et al. 2023). Consequently, microalgae are cultivated and grown directly in the wastewater (Satya et al. 2023). However, various contaminants in the wastewater significantly impact microalgae cultivation. Heavy metals such as lead, cadmium, and copper are toxic to microalgae growth, impeding their essential metabolic processes. Additionally, organic pollutants such as pesticides, industrial chemicals, pathogens, and viruses can cause infection and damage microalgae cells. The instability of pH, temperature, salinity, suspended solids, and low oxygen levels further imposes stress and potential harm to microalgae growth (De Morais et al. 2023).

Although PW contains nitrogen, phosphorus, and trace elements essential for microalgae growth, these elements can sometimes become toxic to the microalgae (Al-Ghouti et al. 2019). Usually, municipal wastewater is deemed suitable for microalgae growth due to its relatively low level of poisonous and harmful substances (Li et al. 2019; Moondra et al. 2020). For example, *Chlorella* sp., *Spirulina* sp., and *Scenedesmus* sp. grew in a fish effluent, showing promising results regarding nutrient uptake and removal efficiency. These microalgae species have converted absorbed nutrients into lipids, polysaccharides, proteins, carbohydrates, and other bioactive compounds within their organelles (Rifna et al. 2023). Furthermore, mixed culture media can enhance algae growth inside municipal wastewater. For example, BG-11 combined with food processing waste powders has significantly increased biomass concentration, lipid, protein, and carbohydrate with 44, 11, 20, and 57% contents, respectively, compared to those grown in BG-11 alone (Peter et al. 2023). BG-11 stands for Blue-green 11 medium, derived from Medium-11, commonly used for growing cyanobacteria. BG-11 medium is not only used for marine microalgae but also freshwater microalgae (Pandey et al. 2023). The main ingredients in BG-11 are sodium nitrate, potassium phosphate, and magnesium sulfate, which play a pivotal role in fostering microalgae growth (Yang et al. 2015).

Since PW contains other toxic elements, considering activated sludge as a pretreatment is noteworthy. Many studies successfully integrated activated sludge with microalgae systems to treat municipal wastewater demonstrating its effectiveness (Arias et al. 2018; Qiao et al. 2020; Qv et al. 2023). The synergistic actions of bacteria and microalgae significantly contributed to reducing the contaminants. For example, an integrated system featuring *Dokdonella* and *Thermomonas* has facilitated nitrogen removal and enhanced phosphorus assimilation (Qv et al. 2023). Moreover, a system with activated sludge has resulted in an increased growth rate of microalgae, with a notable 20% rise in microalgae concentration (Li et al. 2023). Additionally, this system has the advantage of not producing methane or nitrogen oxide gas, unlike other treatment plants, making it an environmentally friendly option (Arias et al. 2018; Qiao et al. 2020; Qv et al. 2023).

A photobioreactor (PBR) can conduct microalgae-based treatment in which microalgae utilizes light equipped with the PBR as a source of energy for microalgae growth. The PBR system also includes an agitator for mixing, which helps mitigate nutrient gradients, enhance mass transfer, and facilitate the separation of gas and liquid culture (Shaikh et al. 2023). One of the advantages of PBR is its ease of construction, maintenance, and cleaning (Peter et al. 2023; Shaikh et al. 2023; Ahmad et al. 2021). Table 2 summarizes recent studies on wastewater treatment using PBRs. It is evident from the table that wastewater treatment in PBRs effectively reduces chemical oxygen demand, phosphorus, nitrogen, and ammonia content.

**Table 2:** Semi-continuous microalgae photobioreactor for wastewater treatment

No.	Type	PBR Dimension	Source of Wastewater	Microalgae	Parameter	Removal efficiency	Reference
1	Thin-layer cascade reactor	Area = 30 m^2^ Depth = 0.04 m	Pig slurry	*Scenedesmus almeriensis*	• Total organic carbon (TOC) • Inorganic carbon (IC) • Total nitrogen (TN)	TOC = 56.9 ± 0.6% IC = 63.9 ± 0.6% TN = 88.6 ± 0.9%	(Zambrano et al. 2023)
2	Bubble column PBR	Volume = 3 L Diameter = 10 cm Height = 50 cm	Agriculture industries	*Scenedesmus* sp. SPP	• COD • TN • Total phosphorus (TP)	COD = 96.2 ± 0.0% TN = 88.2 ± 2.8% TP = 71.5 ± 0.7%,	(Maneechote et al. 2023)
3	Flat panel PBR	Length = 100 cm Width = 20 cm Height = 80 cm	Wetland	*Chlorella vulgaris*	• COD • TN • TP	COD = 39.33% TN = 21.27% TP = 88.10%,	(Zhuang et al. 2023)
4	PBR	Height = 7.5 in Diameter = 6 in	Sewage treatment plant, municipal, slaughterhouse	*Chroococcus* sp.	• COD • NO_3_^-^-N • Ammonia nitrogen (NH_4_^+^ - N) • PO_4_^3−^	COD = 45–72% NO_3_^−^-N NH_4_^+^-N, PO_4_^3−^= 90–98%	(Chawla et al. 2022)
5	PBR	Length = 24 cm Width = 5 cm Height = 35 cm	Synthetic effluent	*Chlorella* sp.	• Nitrogen • Phosphorus	Nitrogen = > 73% Phosphorus = > 90%	(Wang et al. 2022b)
6	Membrane PBR	Volume = 50 L width = 0.4 m length = 0.2 m height = 0.75 m	Pig slurry	*Chlorella vulgaris*	• NH_4_^+^ • PO_4_^3−^ • COD	NH_4_^+^ = 74.55% PO_4_^3−^ = 70.20% COD = 65.85%	(Nguyen et al. 2022)
7	Thin layer + bubble column PBR	Volume = 2400 L + 250 L	Pig slurry	*Nannochloropsis gaditana*	• NH_4_^+^-N	NH_4_^+^-*N* = 63–73%	(Jiménez Veuthey et al. 2022)
8	Raceway pond	Width = 13 cm Height = 26 cm Length = 80 cm	anaerobically digested abattoir effluent	*Scenedesmus*	• NH_4_^+^-N	NH_4_^+^-*N* = 50–85%	(Shayesteh et al. 2023)
9	Airlift PBR	Volume = 1.5 L	eel aquaculture	*Desmodesmus* sp.	• TP • COD • NH_4_^+^-N • TN	TP = 96.1%, COD = 98.0% NH_4_^+^-*N* = 100.0% TN = 97.4%	(Zheng et al. 2023)

Table 3 presents a list of studies that related to PW treatment by microalgae. Several types of algae involved in PW treatment include *Scenedesmus, Chlorella*, and *Nannochloropsis*. The studies listed demonstrate excellent performance in pollutant removal, including chemical oxygen demand (COD), various oils, iron, nitrogen, phosphorous, total organic carbon (TOC), total nitrogen (TN), polycyclic aromatic hydrocarbons (PAHs), total hydrocarbons, phosphorus, copper (Cu), lead (Pb), cadmium, nitrate, and phosphate. Some of the studies involved the addition of nutrients to support treatment, but most did not. Due to the presence of harsh and toxic substances detrimental to microalgae, additional treatment or nutrients are essential. Therefore, microalgae bioremediation needs further exploration and investigation. It can create an environment that nurtures healthy microalgae growth, ensuring the overall success and sustainability of pollutant removal efficiency in PW.

**Table 3:** Available studies on PW treatment by microalgae

No.	Produced water sources	Microalgae strain	Pretreatment/ media supplementation	Pollutant removal efficiency	Ref.
1	Oil field, Saudi Arabia	*Scenedesmus obliquus*	Activated sludge	TOC = 64% TDS = 49.8% Barium = 95% Iron = 52% Manganese = 76%	Current study
2	Oil field, Iraq	*Nanno-* *chloropsis oculate*, *Isochrysis galbana*	Supplement with BG-11 nutrient media	COD = 81% Oil = 72%	(Ammar et al. 2018)
3	Operating site, France	*Nanno-chloropsis oculata*	Supplement with nitrogen phosphorous nutrients	COD = 70% Iron = > 90% NH_4_^+^-*N* = 100%	(Parsy et al. 2020)
4	Petroleum company, Qatar	*Chlorella* sp. *Scenedesmus* sp.	Pretreated with NaOH	TOC = 73% TN = 92%	(Das et al. 2018)
5	Oil field, Brazil	*Nanno-* *chloropsis oculate*	-	Iron = 96.8% PAHs = 94% PHE = 99%	(Marques et al. 2021)
6	Oil and gas facility, USA	*Galdieria sulphuraria*	-	TN = 99.6 ± 0.2% Phosphorus = 74.2 ± 8.5%	(Rahman et al. 2021)
7	Oil field, Algeria	*Chlorella pyrenoidosa*	-	COD = 89.67% TN = 57.14% TP = 75.51% Copper = 73.39% Lead = 72.80% Cadmium = 48.42%	(Rahmani et al. 2022)

To our knowledge, this is the first integrated system comprising activated sludge and microalgae designed for PW treatment. The synergistic potential between these two treatment methods is promising for enhancing PW bioremediation efforts. Our study uses a sequential batch reactor (SBR) and PBR containing *S. obliquus*. The selection of the SBR as the pretreatment medium for PW preceded the application of the PBR. The *Scenedesmus* strain has previously demonstrated promising capabilities in nutrient removal from various wastewater types and has proven effective in biomass accumulation. This study aims to assess the effectiveness of pollutant removal performed by the microalgae in the PBR system after pretreatment by the SBR.

## Methodology

### Microalgae culture preparation

*Scenedesmus obliquus* has been selected as the preferred microalgae species for this study due to its ability to thrive in PW treatment plants (Johnson et al. 2016). *S. obliquus* was precultured in a Bold basal media (BBM) using a 1 L Erlenmeyer flask. The culturing was carried out at room temperature, under an 8-hour photoperiod, and with aeration. Once *S. obliquus* reached the stationary phase with an optical density above 3.0, the culture was transferred to a 13 L photobioreactor (PBR).

The choice of culture media was based on a comparative study of three different media: BG-11, BBM, and HS CHU-10. The study revealed that BBM was the most suitable and effective medium for cultivating *S. obliquus*, making it the optimal choice for this research (Yadav et al. 2023).

### Pretreatment of produced water

The produced water (PW) was pretreated in a sequential batch reactor (SBR) with a volume of 3.3 L (height = 750 mm, diameter = 75 mm). The SBR was filled with activated sludge collected from a local sewage plant. This reactor operates in a semi-continuous mode, which involves a cycle of filling with raw PW, the reaction in the SBR, settling, and effluent withdrawal in a series of sequential phases. The cycle was set to 24 h and adjusted to 6 h throughout the study. The pretreated PW was withdrawn at 50% of the SBR reactor height. This system was controlled using a programmable logic control system. Table 4 shows the operation conditions of the SBR, while Table 5 summarizes the characteristics of PW before and after the SBR treatment.

**Table 4:** Operating condition of the sequential batch reactor (SBR)

Parameters	
Hydraulic retention time (HRT)	6 h
Organic loading rate (OLR)	5.1 kg COD/m^3^/day
Mixed liquor suspended solid (MLSS)	4.0–4.2 g/L
Sludge volume index (SVI)	65–74 mL/g/MLSS

**Table 5:** Characteristics of PW before and after activated sludge pretreatment

Parameters	Raw PW characteristic	Pretreated PW characteristic
pH	8.41	7.63
Salinity (PSU)	7.86	5
Total dissolved solid (ppm)	6795	4583
Total organic carbon (ppm)	630	120
Conductivity (mS/cm)	12.91	9.16
Arsenic (mg/L)	< 0.010	< 0.010
Barium (mg/L)	1.868	1.234
Cadmium (mg/L)	< 0.005	< 0.005
Cobalt (mg/L)	0.005	0.005
Chromium (mg/L)	0.019	0.005
Cooper (mg/L)	0.262	0.063
Iron (mg/L)	0.469	0.155
Manganese (mg/L)	24.00	5.49
Molybdenum (mg/L)	0.010	0.010
Nickel (mg/L)	0.010	0.010
Lead (mg/L)	< 0.010	< 0.010

### Treatment in photobioreactor

The pretreated PW was directed into a 13 L photobioreactor (PBR) (height = 74 cm, diameter = 15 cm) containing *S. obliquus* culture. Initially, 10% of the pretreated PW from the SBR was fed into the PBR, resulting in a 1:9 ratio inside the tank. The PBR has fluorescent lights (18 W, 740 lm), an air diffuser, and a stirrer. The reaction was carried out with an 8-hour photoperiod and at room temperature. The *S. obliquus* was left to acclimatize to their new surroundings. Once *S. obliquus* growth was detected, a sample (effluent) was withdrawn for analysis. Before analysis, the sample was centrifuged at 7500 rpm for 15 min using Centrifuge 5430 (Eppendorf), and the supernatant was collected. Following the first stage (10% PW), a second-stage addition of 25% PW was carried out once *S. obliquus* growth in the PBR entered the exponential phase. The *S. obliquus* was again left to acclimatize, and the subsequent procedures were repeated.

**Fig. 1:**
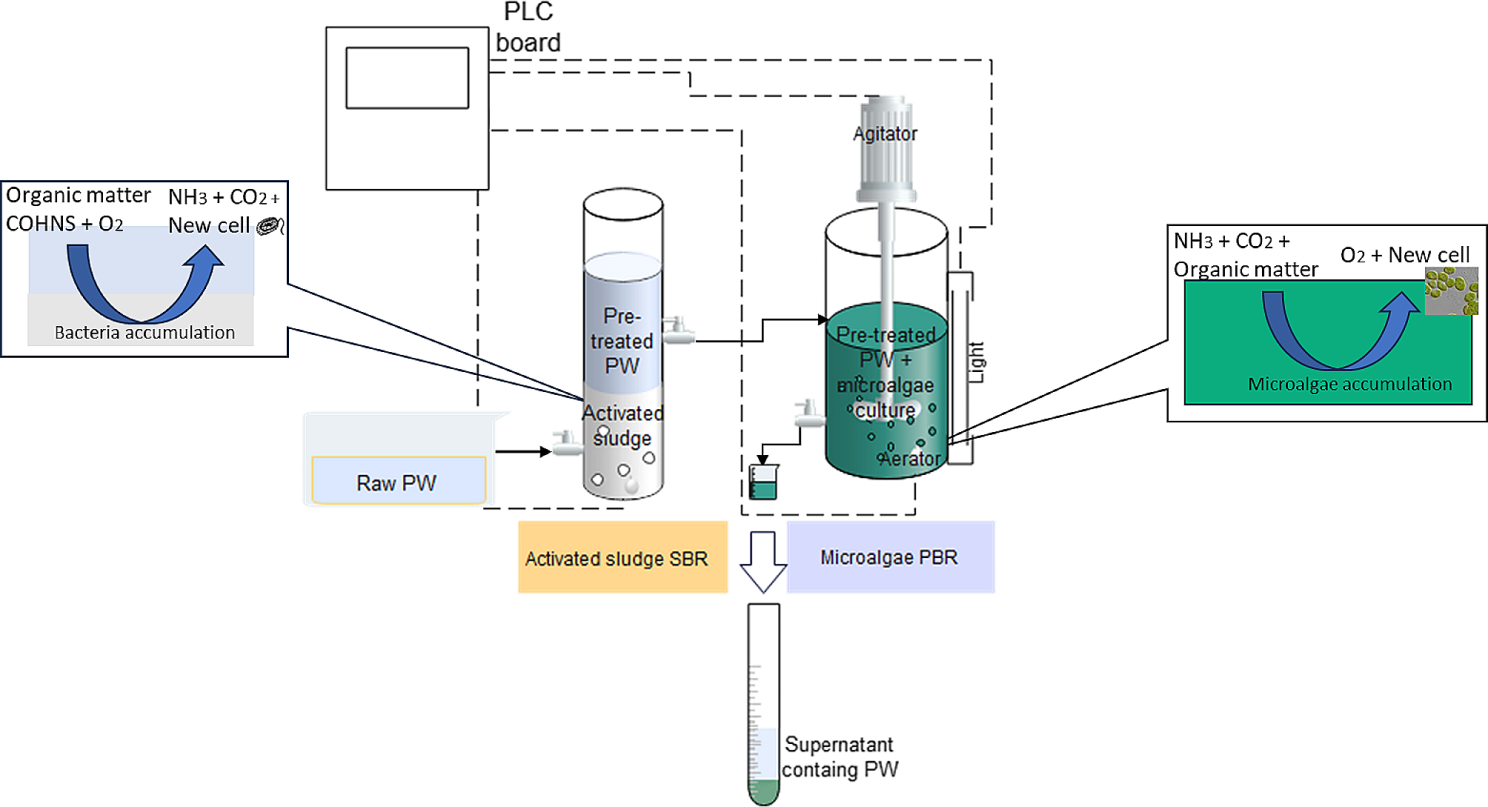
Schematic set up of PW treatment in SBR and PBR with their mechanisms

**Fig. 2:**
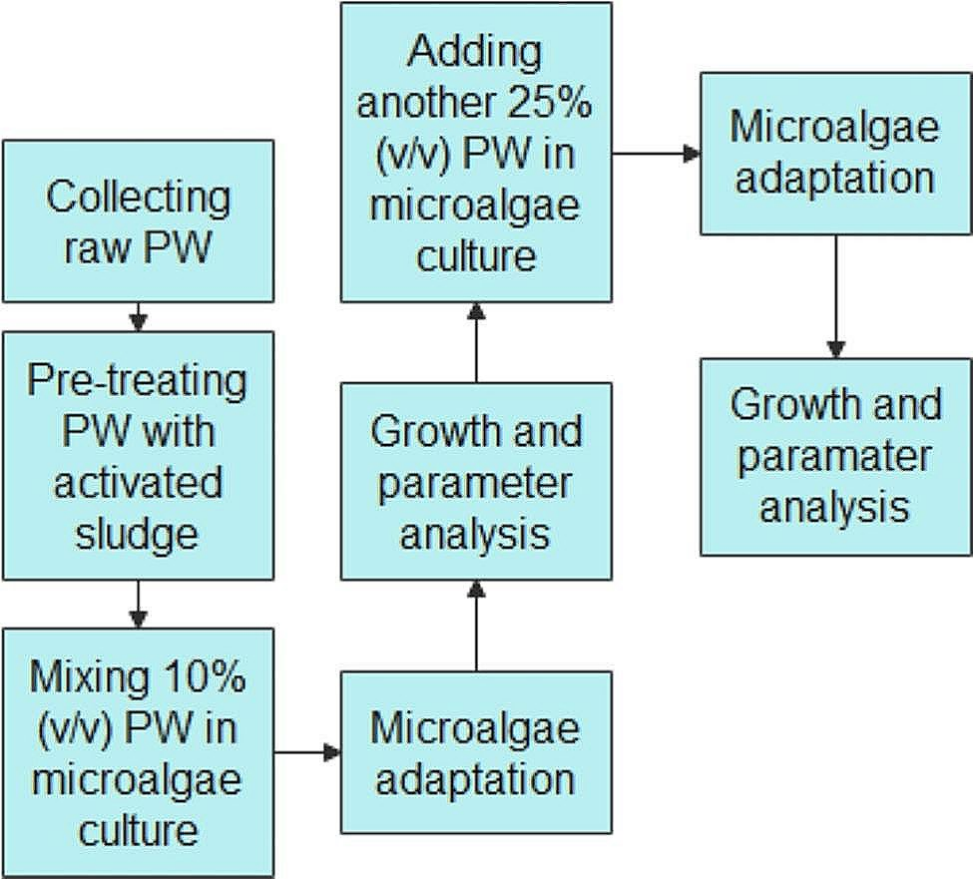
Procedure of integrated PW treatment in SBR and PBR

In the literature, microalgae have been tested across a range of PW concentrations (10–50% v/v), with nutrient supplementation introduced between each PW loading (Das et al. 2018). However, this study diverges from previous research by not involving additional nutrients during the second PW loading. The objective was to evaluate the *S. obliquus*’s ability to remove pollutants solely using the existing nutrients within the wastewater. Figures 1 and 2 depict the experimental setup, potential mechanisms in the reactors, and the overall procedure of PW treatment.

### Microalgae growth

**Fig. 3:**
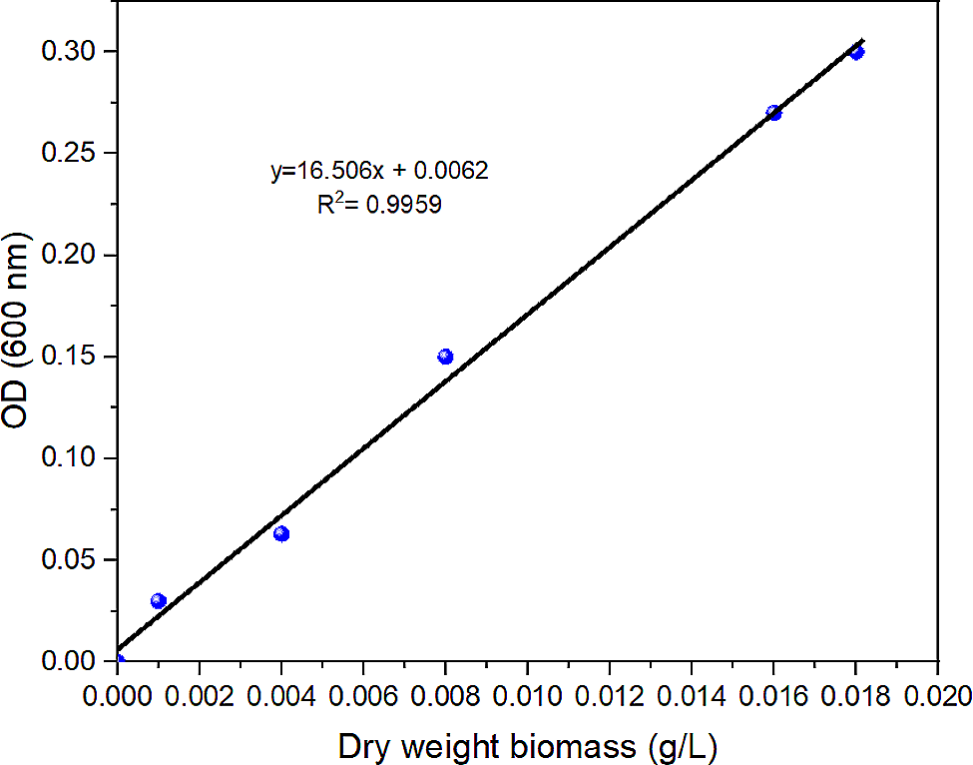
Standard curve of optical density versus microalgae dry weight biomass concentration

The growth of *S. obliquus* in PW was observed by measuring optical density (OD) using ultraviolet-visible spectroscopy (Thermo Scientific) at 600 nm (Ammar et al. 2018). The analysis was conducted in triplicate, and the average reading was recorded accordingly. Then, the ODs were correlated with the concentration of *S. obliquus* cells in g/L dry weight using a calibration curve (Fig. 3). The calibration curve was constructed by measuring the OD values of a serial dilution of *S. obliquus* culture. The data fitted the standard curve with a correlation coefficient of 0.9959. Based on the determined dry weight concentration, the specific growth of *S. obliquus* was calculated using Eq. 1, where X_o_ is the biomass concentration of *S. obliquus* obtained at the beginning of the growth time (t_o_) and X is the biomass concentration at the end of growth time (t).

1 $$\mu =\frac{\text{ln}\frac{X}{{X}_{o}}}{t- {t}_{o}}$$

The methodology for assessing biomass concentration (g/L) through OD and the standard curve has been described elsewhere (Anagnostopoulos et al. 2011; Chaidir et al. 2020; Govindan et al. 2021).

### Analytical method

The effluent samples withdrawn from the PBR were analyzed for their total organic carbon (TOC), salinity, pH, conductivity, total dissolved solids (TDS), and heavy metals. The analysis was conducted in triplicate, and the average reading was recorded accordingly. TOC analysis was performed using a specific TOC analyzer from Shimadzu, while heavy metal analysis was conducted using ICP-mass spectrometry (Perkin Elmer instrument). Heavy metals (barium, iron, manganese) removal efficiency (R) was calculated using Eq. 2, where C_i_ is the initial concentration, and C_f_ is the final concentration of the heavy metals.

2 $$R\left(\%\right)=\frac{{\text{C}}_{\text{i}}\text{-} {\text{C}}_{\text{f}}}{{\text{C}}_{\text{i}}}\times\text{100}$$

The samples’ salinity, pH, conductivity, and TDS were determined using Hanna Instrument water quality probes. The removal efficiency of TDS and conductivity was calculated based on the lowest reading achieved in the PBR.

## Result and discussion

### The growth of microalgae in the PW

**Fig. 4:**
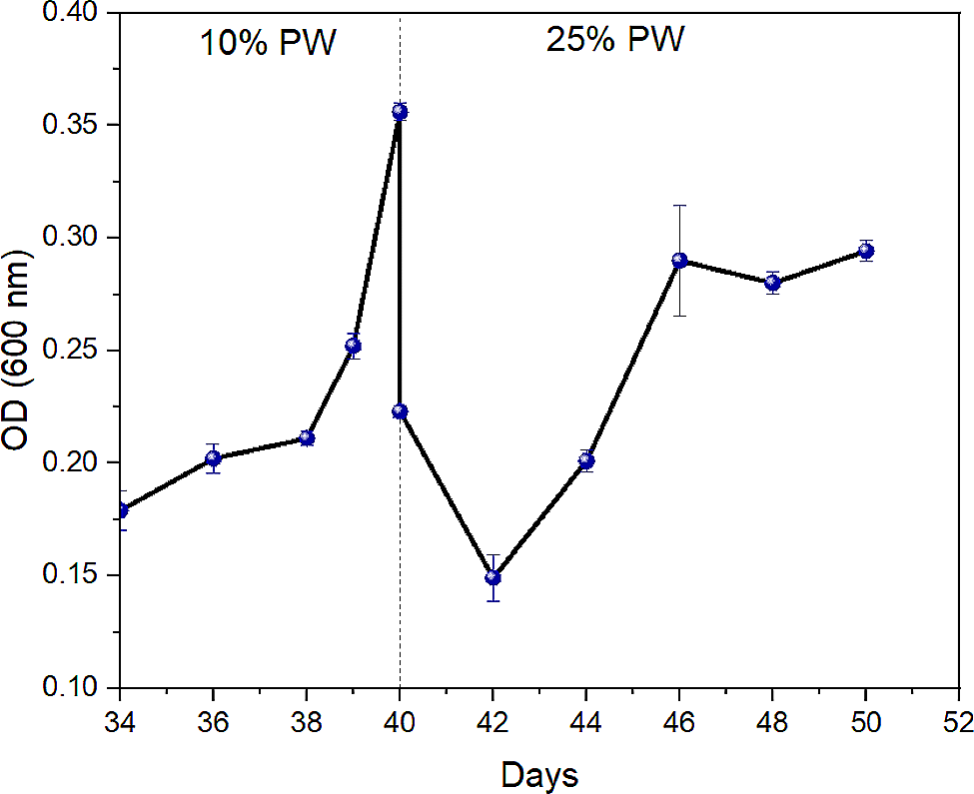
Microalgae growth performance in PBR

The growth performance of *S. obliquus* in the PBR was assessed in two stages: 10% PW and 25% PW through optical density (OD) reading. Initially, the OD of *S. obliquus* culture was 0.29, and it decreased with the increase in days until Day 34 (data not shown in the graph). From Day 34 to 38, slight increments in the OD reading were recorded (Fig. 4), indicating that the microalgae had entered a lag phase. Subsequently, an exponential phase was observed on Day 40, indicated by an increased OD at 0.36. Another 25% of PW was added to the PBR following this observation. The OD reading decreased again due to the dilution effect caused by the said PW, which continued to decline for 2 days after the addition. This was because *S. obliquus* was acclimatizing and trying to adapt again to the PW. Notably, no lag phase was observed after the second time adaptation period. *S. obliquus* grew exponentially on Day 42 and reached the stationary phase by Day 46. *S. obliquus* growth profile is consistent with the results reported in other studies related to microalgae growth in wastewater (Kumar et al. 2020; Japar et al. 2021; Rahmani et al. 2022).

Adding 25% PW seemed more detrimental to *S. obliquus* growth as the highest achieved OD was only 0.29, compared to 0.36 in the first stage with 10% PW. This suggests that *S. obliquus* growth would be hindered above 10% PW loading. Similarly, literature reports that C. vulgaris exhibited significant growth at 5% and 10% PW concentrations but experienced growth reduction at 20% PW, while *G. sulphuraria* strain demonstrated optimal growth at 20% PW concentration (Rahman et al. 2021). These observations collectively highlight the varying tolerance levels of different microalgae strains to PW concentrations. The shortest adaptation period was recorded after the second stage of PW loading (25% v/v), which was only two days, compared to 34 days at the first stage of PW loading. This indicates that increasing the PW concentration does not significantly affect *S. obliquus* adaptation, suggesting that *S. obliquus* can endure the environment. Under severe stress, adaptation can occur by acquiring beneficial phenotypes via random genomic mutations and subsequent positive selection (Sun et al. 2018). During the adaptation period, microalgae growth can be inhibited, and cell numbers may be reduced due to toxicity. This toxicity results in the degradation of intracellular compounds, such as photosynthetic pigments, leading to a reduction in cell size (Cavalcanti Pessôa et al. 2022).

Adaptation periods and lag phases significantly impact hydraulic retention time (HRT), a crucial factor in biological treatment processes. Optimal HRT is essential because it influences the overall efficiency of the treatment. A prolonged lag phase can lead to an extended HRT, which lengthens the treatment process and may cause nutrient depletion, ultimately resulting in microalgae death. Conversely, an excessively short HRT is impractical, as it needs to provide more time for microalgae to adapt and effectively remove pollutants. Therefore, achieving an optimal HRT is critical for making large-scale treatment more feasible. Reducing the time required for wastewater to pass through the treatment system can enhance efficiency and scalability (Soroosh et al. 2023). In this study, the HRT of the PBR was shorter, with a recorded HRT of 6 days (from the adaptation period to the exponential growth phase) before reaching the stationary phase. Typically, microalgae are harvested upon reaching the stationary phase, at which point microalgae usually achieve the highest biomass and lipid content (Wang et al. 2022a). Despite the hazardous nature of PW, the pretreatment with activated sludge facilitated the HRT in PBR comparable to that of microalgae treated in less toxic municipal wastewater, which typically ranges from 2 to 8 days (Zhang et al. 2020).

### Total organic carbon removal

**Fig. 5:**
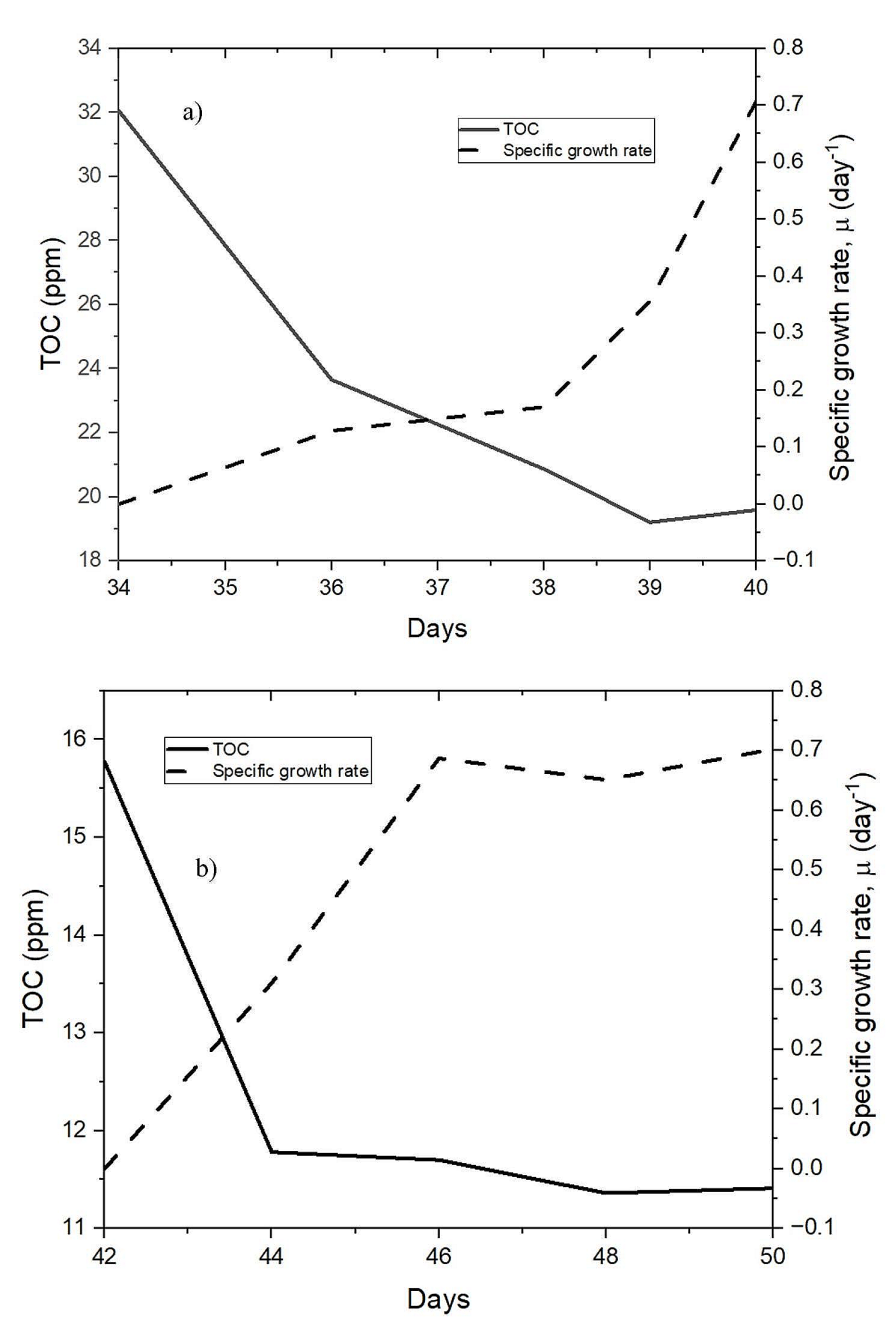
(**a**) TOC removal with 10% PW added (**b**) TOC removal with 25% PW added

The total organic carbon (TOC) reading of the sample withdrawn from the PBR after Day 34 was recorded at 32.1 ppm. Over six days, the TOC reading was further reduced to 19.6 ppm (Fig. 5(a). Comparing this with the specific growth rate achieved in the PBR, it was observed that the TOC decreased as the growth increased. Microalgae require organic carbon for their growth and development. While they can utilize inorganic carbon sources such as carbon dioxide (CO_2_) for photosynthesis, they also rely on organic carbon as an essential component of their nutritional requirements (Das et al. 2018). Some microalgae utilize inorganic and organic compounds, and some thrive solely on CO_2_ during photosynthesis. Notably, around 50% of the carbon content in microalgae comprises organic compounds (Lee and Lee 2002).

Subsequently, after adding 25% PW to the PBR, the TOC concentration was further reduced from 16 ppm to 11.4 ppm (Fig. 5(b). This decrease in TOC after the second stage loading resulted in a total removal efficiency of 64%. Throughout bioremediation process, microalgae absorbed organic carbon, even during its lag phase. During this phase, microalgae reduced high TOC levels as they adapted to the environment and initiated growth. Interestingly, the organic carbon content in microalgae is comparatively lower during the exponential phase. This is attributed to changes in metabolic priorities during different growth stages (Xian et al. 2022).

Organic content in PW originates from various sources within the oil and gas production process. Primarily, it consists of alcohols, with methanol and non-methane hydrocarbons being particularly abundant. These hydrocarbons typically fall within the C6-C9 range of alkanes and aromatic hydrocarbons. Lighter hydrocarbons tend to volatilize from PW before reaching storage or disposal ponds (Lyman et al. 2018). Other organic compounds include polycyclic aromatic hydrocarbons (PAHs), phenolic compounds, glycol ethers, and cyclic ketones (Varonka et al. 2020).

Mixotrophs are microalgae that consume organic carbon. The addition of organic carbon, alongside CO_2_, enhances the production of biomass and lipids in these microalgae (Vasistha et al. 2023). In this study, the mixotrophic growth ability of *S. obliquus* is demonstrated by the increase in its specific growth rate. Most *Scenedesmus* species grow well under mixotrophic conditions. For example, *Scenedesmus quadricauda* exhibited a higher biomass yield when grown in mixotrophic conditions than its heterotrophic mode, aided by monochromatic illumination from light-emitting diodes (Korozi et al. 2023). Similarly, *Scenedesmus parvus* achieved high biomass and demonstrated 81% COD and 19% BOD removal efficiency when cultivated in a mixotrophic mode (Ooi et al. 2023). *S. obliquus* (iAR632) has recently been studied for its biosynthetic pathways, revealing that this strain comprises 1467 reactions, 734 metabolites, and 632 genes. Predictions and experimental observations indicated a 3.8-fold increase in biomass and almost 4-fold higher lipid content under mixotrophic conditions than other trophic modes (Ray et al. 2023).

### Total dissolved solids, electrical conductivity, pH, and salinity

**Fig. 6:**
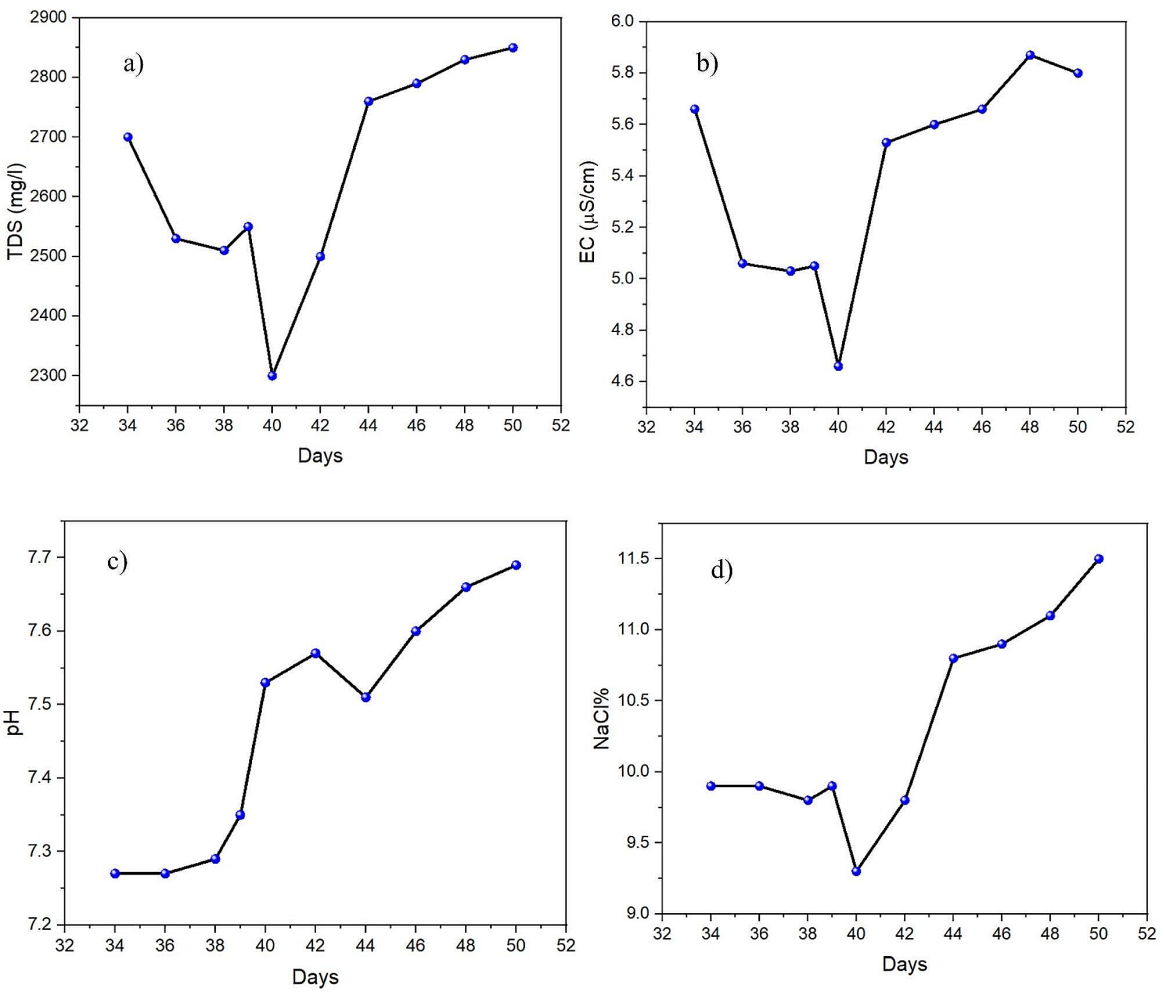
(**a**) Total dissolved solids (TDS), (**b**) electrical conductivity (EC), (**c**) pH, and (**d**) NaCl% of sample from PBR

The total dissolved solids (TDS) level of effluent samples withdrawn from PBR showed a decrease in TDS after 10% PW loading (Fig. 6 (a). The TDS removal efficiency achieved in this study was higher than others, reaching 49.8%. In contrast, a previous study reported TDS removal efficiencies of 46.42% for non-filtered water and 48.71% for filtered wastewater in microalgae-based treatment (Moondra et al. 2020). However, upon the second loading (25% of PW), the TDS levels ceased to decrease and were even slightly higher than the TDS reading at 10% loading. Besides that, the electrical conductivity (EC) of the samples decreased from 5.66 mS/cm to 4.66 mS/cm after the first PW loading (10%) (Fig. 6 (b). The EC reduction continued gradually, reaching 49.1% removal, higher than the reported percentage, 46.43% for non-filtered and 48.47% for filtered wastewater. The EC result was consistent with the result observed in TDS removal. This correlation arises from the interrelation between these two parameters. An increase in TDS leads to a corresponding increase in EC, as the rise in EC is attributed to the presence of ionic species within the TDS (Rusydi 2018).

The effluent samples withdrawn from the PBR had pH readings ranging from 7.2 to 7.6 (Fig. 6 (c). The raw PW’s pH value was initially highly alkaline, at 8.4. In contrast, PW from other reported sources had a pH of 4.17, indicating acidic wastewater. NaOH was added to the PW to raise the pH to 7.1 and facilitate the removal of TOC (Das et al. 2018).

Furthermore, the salinity measurement (NaCl%) of the effluent samples showed a slightly consistent reading, indicating no desalination had occurred across the PBR. After the second PW loading (25%), the salinity increased further (Fig. 6 (c). Although a previous study reported that *S. obliquus* could remove salt from saline water (Wei et al. 2020), this was not observed here. The study reported that the removal mechanism involved absorption and adsorption, with adsorption playing a more significant role. Longer contact times resulted in increased adsorption. The study also suggested that different functional groups in *S. obliquus* cells can combine with Na^+^ and Cl^−^ ions. Adding NaCl promoted the synthesis of free and ester-type xanthophylls. Salt stress synergistically activates algal cells’ carotenogenesis, leading astaxanthin’s esterification (Aburai et al. 2015). However, in this study, the constant salinity was believed to be due to the toxic environment of PW because microalgae do not adsorb or absorb salts when under stress (Gan et al. 2016).

### Heavy metals removal

**Fig. 7:**
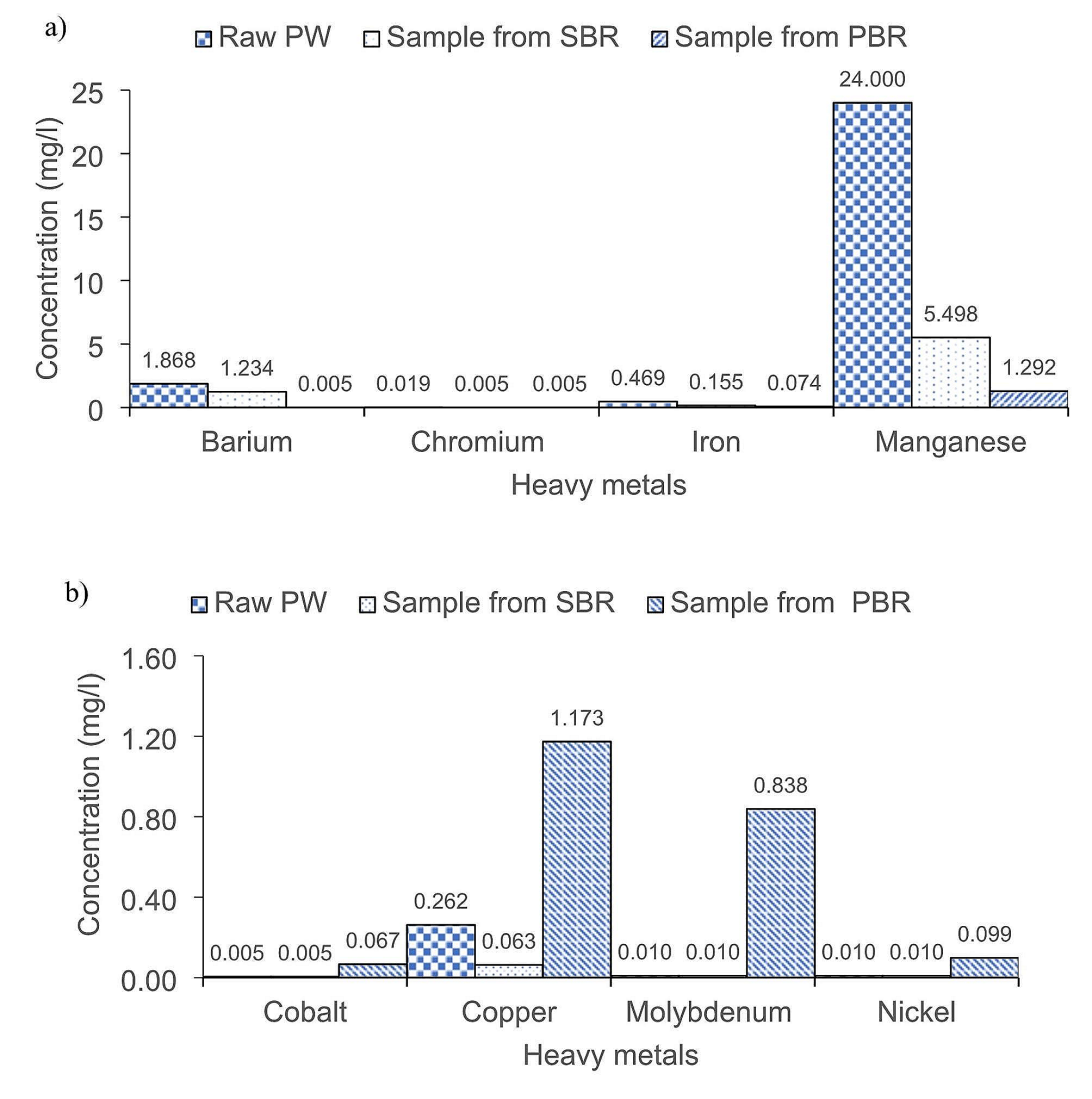
(**a**) Reduction of heavy metals and (**b**) increment of heavy metal contents in samples from SBR and PBR

The analysis of heavy metals in the raw PW used in the study revealed the absence of carcinogenic heavy metals, including arsenic, mercury, cadmium, and lead. As shown in Fig. 7 (a), *S. obliquus* further reduced heavy metals, including barium, iron, and manganese, with total removal efficiencies of 95% for barium, 76% for manganese, and 52% for iron. There was a significant reduction in manganese probably because manganese is one of the essential micronutrients for microalgae growth. Low manganese concentration (0.1–0.3 mg/L) can decrease algae growth rate (Liu et al. 2018). The reduction of heavy metal ions by microalgae can occur through biosorption on the extracellular polymeric substances of the microalgae cells and ion exchange, where heavy metal ions with charges are absorbed onto the oppositely charged biomass surface (Leong and Chang 2020). The microalgae cell walls also can block toxic heavy metals (Xiao et al. 2023).

However, the results showed increased heavy metals, specifically copper and molybdenum (Fig. 7 (b). The increase was likely due to media use during *S. obliquus* cultivation in the PBR. As mentioned, *S. obliquus* culture underwent initial cultivation in the Bold Basal medium (BBM) before being transferred into the PBR. BBM media contains trace metals such as cobalt, copper, iron, manganese, molybdenum, zinc, and selenium, essential for microalgae growth. A study has reported that copper and molybdenum levels in a standard BBM media were 8.82 and 0.71 mg/L, respectively (Alfiarty 2018). Generally, copper serves as a growth factor for microalgae, while molybdenum and nickel play essential roles in nitrogen assimilation. Under certain conditions, molybdenum could be excreted from the microalgae cells to regulate the internal concentration of this element (Tejada-Jimenez et al. 2023). Therefore, the increase of copper and molybdenum observed in the heavy metal determination was likely due to the BBM media utilized in this experiment. Furthermore, pH level plays a critical factor in affecting the ability of *S. obliquus* to remove copper and molybdenum. In this study, the pH level of *S. obliquus* culture and PW inside the PBR was above 7, whereas the optimum pH for these heavy metals adsorption by microalgae is below 7 (Saavedra et al. 2018; Liu et al. 2021; Novák et al. 2020; Urrutia et al. 2019; Tambat et al. 2023).

## Conclusion

Integrating semi-continuous SBR with a batch PBR system holds significant potential for future PW treatment. This combination allows for a reduction in hydraulic retention time (HRT), enhancing the overall efficiency of the treatment process. Organic carbon content in PW can be effectively reduced as it is consumed by *S. obliquus* during the bioremediation process. Additionally, the content of heavy metals in PW can be reduced, although the media and pH levels may limit the extent of reduction for some metal levels. Overall, this integrated approach offers a promising solution for improving the quality of PW treatment through bioremediation methodologies.

## Data Availability

The datasets used and/or analyzed during the current study are available from the corresponding author upon reasonable request.

## Abbreviations

EC: Electrical conductivity
COD: Chemical oxygen demand
BOD: Biochemical oxygen demand
HRT: Hydraulic retention time
OD: Optical density
PBR: photobioreactor
PLC: Programmable logic control
PW: Produced water
TDS: Total dissolved solids
TOC: Total organic carbon
