# Correction: Produced water treatment by semi-continuous sequential bioreactor and microalgae photobioreactor

**DOI:** 10.1186/s40643-024-00778-0

**Published:** 2024-07-01

**Authors:** Nur Farahah Mohd Khairuddin, Nadeem Khan, Saravanan Sankaran, Wasif Farooq, Irshad Ahmad, Isam H. Aljundi

**Affiliations:** 1 Membranes and Water Security IRC, King Fahd University of Petroleumand Minerals (KFUPM), Dhahran, Saudi Arabia; 2 Bioengineering Department, King Fahd University of Petroleumand Minerals (KFUPM), Dhahran, Saudi Arabia; 3 Chemical Engineering Department, King Fahd University of Petroleumand Minerals (KFUPM), Dhahran, Saudi Arabia

**Correction: Bioresources and Bioprocessing (2024) 11:56**

10.1186/s40643-024-00775-3

Following publication of the original article, the authors would like to correct the acknowledgments and funding section.

The sentence currently reads:

**Acknowledgements** The first author would like to acknowledge the support received from the IRCMembranes and Water Security, Bioengineering Department, and Chemical Engineering Department of King Fahd University of Petroleum and Minerals (KFUPM). This acknowledgment also goes to the Interdisciplinary Research Center for Refining and Advanced Chemicals (CRAC) at King Fahd University of Petroleum and Minerals (KFUPM) for funding project INMW2304.

**Funding** Interdisciplinary Research Centre for Refining and Advanced Chemicals (CRAC), KFUPM. Funding no. INMW2304.

The sentence should read:

**Acknowledgements** The first author would like to acknowledge the support received from the Membranes and Water Security IRC, Bioengineering Department, and Chemical Engineering Department of King Fahd University of Petroleum and Minerals (KFUPM) **as well as the funding for project INMW2304 provided by the Membranes and Water Security IRC**.

**Funding** Membranes and Water Security IRC, KFUPM. Funding no. INMW2304.

The original article has been corrected.

